# Transposon-mediated directed mutation controlled by DNA binding proteins in *Escherichia coli*

**DOI:** 10.3389/fmicb.2014.00390

**Published:** 2014-08-01

**Authors:** Milton H. Saier, Zhongge Zhang

**Affiliations:** Division of Biological Sciences, Department of Molecular Biology, University of California at San DiegoLa Jolla, CA, USA

**Keywords:** directed mutation, transposable genetic element, transposon, insertion sequence, gene activation, *glpFK* operon

## Introduction

It is a basic principle of genetics that the likelihood of a particular mutation occurring is independent of its phenotypic consequences. The concept of directed mutation, defined as genetic change that is specifically induced by the stress conditions that the mutation relieves (Cairns et al., 1988), challenges this principle (Foster, 1999; Rosenberg, 2001; Wright, 2004). The topic of directed mutation is controversial, and its existence, even its *potential* existence, as defined above, has been altogether questioned (Roth et al., 2006).

Part of the justifiable skepticism concerning directed mutation resulted from experiments that were purported to demonstrate this phenomenon, but were subsequently shown to be explainable by classical genetics (Roth et al., 2006). Mutation rates vary with environmental conditions (e.g., growth state) and genetic background (e. g., mutator genes), a phenomenon known as “adaptive” mutation (Wright, 2004; Foster, 2005), but this does not render the mutation “directed.” To establish the principle of directed mutation, it is necessary to show that the adaptive mutation is “directed” to a specific site, characterize the mechanism responsible, identify the proteins involved, and provide the evolutionary basis for its appearance.

One frequently encountered type of mutation results from the hopping of transposable genetic elements, transposons, which can activate or inactivate critical genes (Mahillon and Chandler, 1998; Chandler and Mahillon, 2002). For example, activation of the normally cryptic β-glucoside (*bgl*) catabolic operon in *E. coli* can be accomplished by insertion of either of two insertion sequences, IS1 or IS5, upstream of the *bgl* promoter (Schnetz and Rak, 1992).

**Figure 1:**
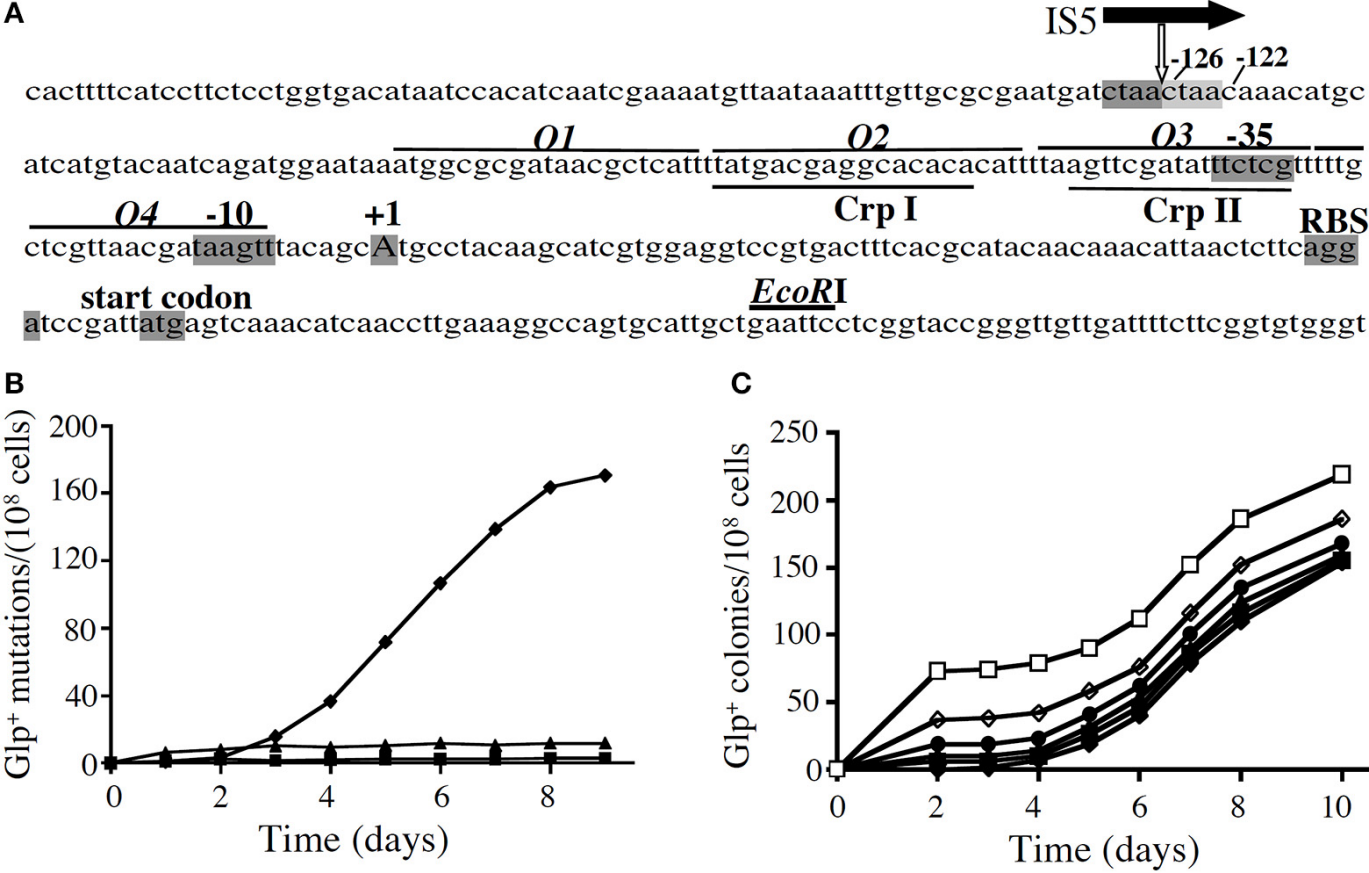
**The appearance of Glp^+^ mutations in a *crp* genetic background. (A)** The *glpFK* promoter region. The transcriptional initiation site (+1), the −10 and −35 hexamers of the promoter, the ribosome binding site (RBS) and the start codon for *glpF* are shaded. The GlpR binding sites (*O1–O4*; lines above the sequence) and CRP binding sites (CrpI and CrpII; lines below the sequence) are also shown. The location of the IS5 element upstream of the promoter is indicated by the vertical white arrow below the horizontal black arrow representing IS5. The two 4-nucleotide direct repeats (ctaa), caused by IS5 insertion, are shaded dark and light gray, respectively. **(B)** Glp^+^ mutations in a *crp* genetic background in various media: solid M9 minimal media + 1% glycerol (♦), 0.01% glucose (■), or 1% sorbitol (▲). *crp* cells from an overnight culture (from a single colony) were applied onto agar plates, and the plates were incubated at 30°C. On glycerol minimal plates, mutations were determined by the presence of colonies on the plate. On sorbitol and glucose plates, mutations were determined by washing all cells off the plates and measuring colony formation both on LB plates (total cells) and on minimal glycerol plates (Glp^+^ cells) after 36–48 h of growth. **(C)** The same minimal glycerol agar plates, where the *crp* mutant cells were plated together with various numbers of *crp* Glp^+^ cells. Numbers of *crp* Glp^+^ cells were: (□, 72; ◊, 38;●, 19;▲, 10;■, 5; and ♦, 0). These cells were mixed with *crp* cells and then applied onto M9 glycerol agar plates.

The *E. coli* glycerol (*glp*) regulon consists of five operons, two of which (*glpFK* and *glpD*) are required for aerobic growth on glycerol (Lin, 1976). Both operons are subject to negative control by the DNA-binding *glp* regulon repressor, GlpR (Zeng et al., 1996), which also binds glycerol-3-phosphate, the inducer of the *glp* regulon. The *glpFK* operon is additionally subject to positive regulation by the cyclic AMP receptor protein (CRP) complexed with cyclic adenosine monophosphate (cAMP; Freedberg and Lin, 1973; Campos et al., 2013), although *glpD* is not appreciably subject to regulation by this mediator of catabolite repression (Weissenborn et al., 1992). The *glpFK* regulatory region contains four GlpR binding sites, *O1–O4*, and two CRP binding sites, CrpI and CrpII, which overlap *O2* and *O3*, respectively, (Figure 1A). The strong CRP dependency of *glpFK* transcription is reflected by the fact that *crp* and *cya* (adenylate cyclase) mutant cells are unable to utilize glycerol (Zhang and Saier, 2009a). We have found that binding of GlpR and the cAMP-CRP complex to the *glpFK* upstream control region negatively influences IS5 hopping specifically into the single site that strongly activates *glpFK* promoter activity (Zhang and Saier, 2009a, unpublished observations).

## Glp^+^ mutations in a *crp* genetic background

When *crp* cells were incubated on solid glycerol minimal medium, Glp^+^ colonies appeared. We tested the growth of a *crp* Glp^+^ strain on glycerol in defined liquid medium. The growth rate was greater than that of wild type (*wt*) *E. coli* (Zhang and Saier, 2009b).

The relative rates of Glp^+^ mutation were determined in minimal and complex media. On glycerol plates, colonies first appeared after about 3 days (Figure 1B) although wild type (*wt*) and *crp* Glp^+^ *E. coli* cells formed visible colonies in <2 days (Figure 1C). When the same cells were plated as before, but small numbers of *crp* Glp^+^ mutant cells were included with the *crp* cells before plating, colonies appeared from the *crp* Glp^+^ cells within 2 days, and new Glp^+^ mutants arose at the same rate as before (Figure 1C). This experiment showed that the mutants that arose on the plates had not existed in the cell population when initially plated. Thus, the Glp^+^ mutants arising from *crp* cells on these plates arose during incubation on the plate, and no growth inhibitor was present. The rate of mutation on glycerol plates proved to be 10 times higher than in minimal sorbitol or complex LB medium, and it was over 100 times higher than in glucose-containing medium (Zhang and Saier, 2009a).

Mutation proved to be due to IS5 hopping to a discrete site, between base pairs 126 and 127 upstream of the transcriptional start site, and always in the same orientation. The increase in mutation rate was specific to the *glpFK* operon and did not occur in three other operons examined (Zhang and Saier, 2009a). Only the downstream 177 bp region of IS5 was required for activation of the *glpFK* operon, and this proved to be due to the presence of a permanent bend and an overlapping IHF binding site, each of which was responsible for half of the activation (Zhang and Saier, 2009b). This mechanism of activation (but not the actual transposon hopping event), presumably involving DNA looping, could also be demonstrated for the lactose (*lac*) operon in a *crp* genetic background of *E. coli* (Zhang and Saier, 2009b).

## Dependency of the Glp^+^ mutation rate on GlpR

Glycerol is phosphorylated by glycerol kinase (GlpK) to glycerol-3-phosphate which binds to and releases GlpR from its operators (Lin, 1976). When GlpR dissociates from its operators, due to glycerol-3-phosphate binding, a conformational change could be transmitted through the DNA, promoting insertion of IS5 at the target CTAA site upstream of the *glpFK* promoter. In other words, GlpR binding might have two functions: repression of gene expression and suppression of IS5 transposition to the upstream activating site.

To test this possibility, the *glpR* gene was deleted, and the rates of appearance of Glp^+^ mutations in the *crp glpR* double mutant background were measured in the absence and presence of glycerol. The numbers of Glp^+^ cells arising was 10-fold higher in the *crp glpR* double mutant than in the *crp* mutant when glycerol was absent. In the presence of glycerol, the loss of GlpR was without effect. Thus, deletion of *glpR* is equivalent to the inclusion of excess glycerol in the growth medium. High level overexpression of *glpR* decreased mutation rate to background levels (Zhang and Saier, 2009a). Clearly, GlpR regulates the increased rate of IS5-mediated insertional activation of the *glpFK* promoter by glycerol.

## GlpR operators differentially control *glpFK* expression and Glp^+^ mutation rate

There are four GlpR binding sites, *O1–O4*, in the upstream *glpFK* operon regulatory region (see Figure 1A), identified by DNA footprinting (Freedberg and Lin, 1973; Zeng et al., 1996). We mutated the far upstream site (*O1*) and the far downstream site (*O4*) so they no longer could bind GlpR, and compared the effects on *glpFK* expression using a *lacZ* reporter gene fusion construct vs. mutation rate to Glp^+^ during growth in LB medium. Mutation of *O4* increased *glpFK* operon expression about 5-fold although mutation of *O1* was almost without effect (Zhang and Saier, 2009a). By contrast, loss of *O1* yielded a 7-fold increase in mutation rate although loss of *O4* had only a 2-fold effect on mutation rate. We confirmed that IS5 was always in the same position and orientation (Zhang and Saier, 2009a). Thus, *O1* primarily controls the rate of IS5 insertion into the activating site, while *O4* primarily controls *glpFK* expression.

## Control of IS5-mediated *glpFK* operon activation by the cAMP-CRP complex

As noted above, IS5-mediated activation of the *glpFK* promoter was observed in a *crp* genetic background. Initial attempts in our laboratory and elsewhere to isolate such mutants in a wild type genetic background proved unsuccessful (Ibarra et al., 2002; Honisch et al., 2004; Fong et al., 2005; Zhang and Saier, 2011, unpublished observations; Cheng et al., 2014). Since *crp* mutants are not found in nature, this brought into question the suggestion that our discovery of directed mutation in a *crp* mutant of *E. coli* was relevant to the wild type situation, and hence whether it had actually evolved under the pressures of natural selection.

We consequently undertook a systematic analysis of the cAMP dependency of IS5-mediated *glpFK* activation to understand why *crp* mutants but not *wt* cells gave rise to IS5-activated mutants. The *cya* gene, encoding the cAMP biosynthetic enzyme, adenylate cyclase (Cya), was deleted, and as expected, IS5-mediated *glpFK* activation was observed at high frequency. When sub-mM concentrations of cAMP were added to the growth medium, the frequency of these mutations was drastically reduced; 1 mM exogenous cAMP essentially prevented the appearance of these mutants. When *glpR* was deleted in the *cya* genetic background, the frequencies of IS5 insertion increased by about 20-fold (Zhang and Saier, unpublished observations).

These observations led us to experiment with *wt E. coli* cells. Glycerol utilization in these cells is strongly inhibited by the presence of the non-metabolizable glucose analogs, 2-deoxyglucose and α-methyl glucoside, which also lower cytoplasmic cAMP levels by inhibiting adenylate cyclase activity (Saier and Feucht, 1975; Castro et al., 1976; Saier et al., 1976; Feucht and Saier, 1980; Saier, 1989). Wild type cells were therefore plated on minimal salts medium containing 0.2% glycerol and 0.1% 2-deoxyglucose or 0.5% α-methyl glucoside. Not surprisingly, IS5 insertional directed mutants could be isolated under these conditions (Zhang and Saier, unpublished results). Abolition of the CRP binding sites in the *glpFK* upstream regulatory region greatly enhanced the frequency of these mutants in an otherwise *wt* genetic background, even in the absence of a non-metabolizable glucose analog, showing that binding of the cAMP-CRP complex to the *glpFK* control region negatively regulates IS5 insertion. These experiments showed that directed mutation is negatively regulated by binding of both the glycerol repressor, GlpR, and the cAMP-CRP complex to the *glpFK* control region. This explains why IS5 insertion into the activating site of the *glpFK* regulatory region in wild type cells depends on both high glycerol and low cyclic AMP concentrations.

## Directed mutations promoting expression of other operons in *E. coli*

Following the reports of Zhang and Saier (2009a,b) cited above, Wang and Wood (2011) demonstrated directed mutation of the operon (*flhDC*) encoding the flagellar transcriptional master switch in *E. coli*, FlhDC. IS5 insertion into the upstream control region of the *flhDC* operon was responsible. Although the mechanism was not established, the frequency of IS5 insertion clearly increased under swarming conditions in soft agar compared to growth in liquid medium or on solid agar plates where swarming does not occur (Wang and Wood, 2011). We have confirmed and extended their results (Zhang et al., 2013). Moreover, preliminary results suggest that the *E. coli fuc* (fucose; propanediol) operon may also be subject to IS5-mediated directed mutation (Zhang and Saier, 2011; Zhang et al., 2013). It seems that transposon-mediated directed mutation will prove to be important to microbial evolution, and possibly to that in other organisms, partly accounting for the prevalence of these genetic elements in virtually all living organisms.

## Conclusions and perspective

Directed mutation has been defined as genetic change that is specifically induced by the stress conditions that the mutation relieves, but until recently, in no case had such a mechanism been established. We have demonstrated that mutations in the *glpFK* control region, allowing growth of *E. coli crp* or *cya* mutants on glycerol plates, or *wt* cells on glycerol plus 2-deoxyglucose or α-methyl glucoside plates, are specifically induced by the presence of these compounds. The glycerol regulon repressor, GlpR, which binds to its four operators (*O1–O4*) in front of the *glpFK* operon (Figure 1A) and is displaced from these sites when α-glycerol phosphate binds allosterically to GlpR (Zeng et al., 1996), not only controls gene expression, but also controls mutation rate. In this case, GlpR binding negatively influences both *glpFK* operon expression and operon activation by IS5. Our results established that binding of GlpR to *O4*, which overlaps the −10 promoter region, primarily controls gene expression, while binding to *O1* primarily controls IS5 hopping into the specific CTAA site, between 127 and 122 base pairs upstream of the *glpFK* transcriptional start site, that activates the *glpFK* promoter. Binding of the cAMP-CRP complex to its two binding sites overlapping *O2* and *O3* also blocks IS5 hopping to the activating site. This dual mechanism may involve changes in DNA conformation or supercoiling. The results serve to dissociate two functions of both GlpR and CRP.

**Figure 2:**
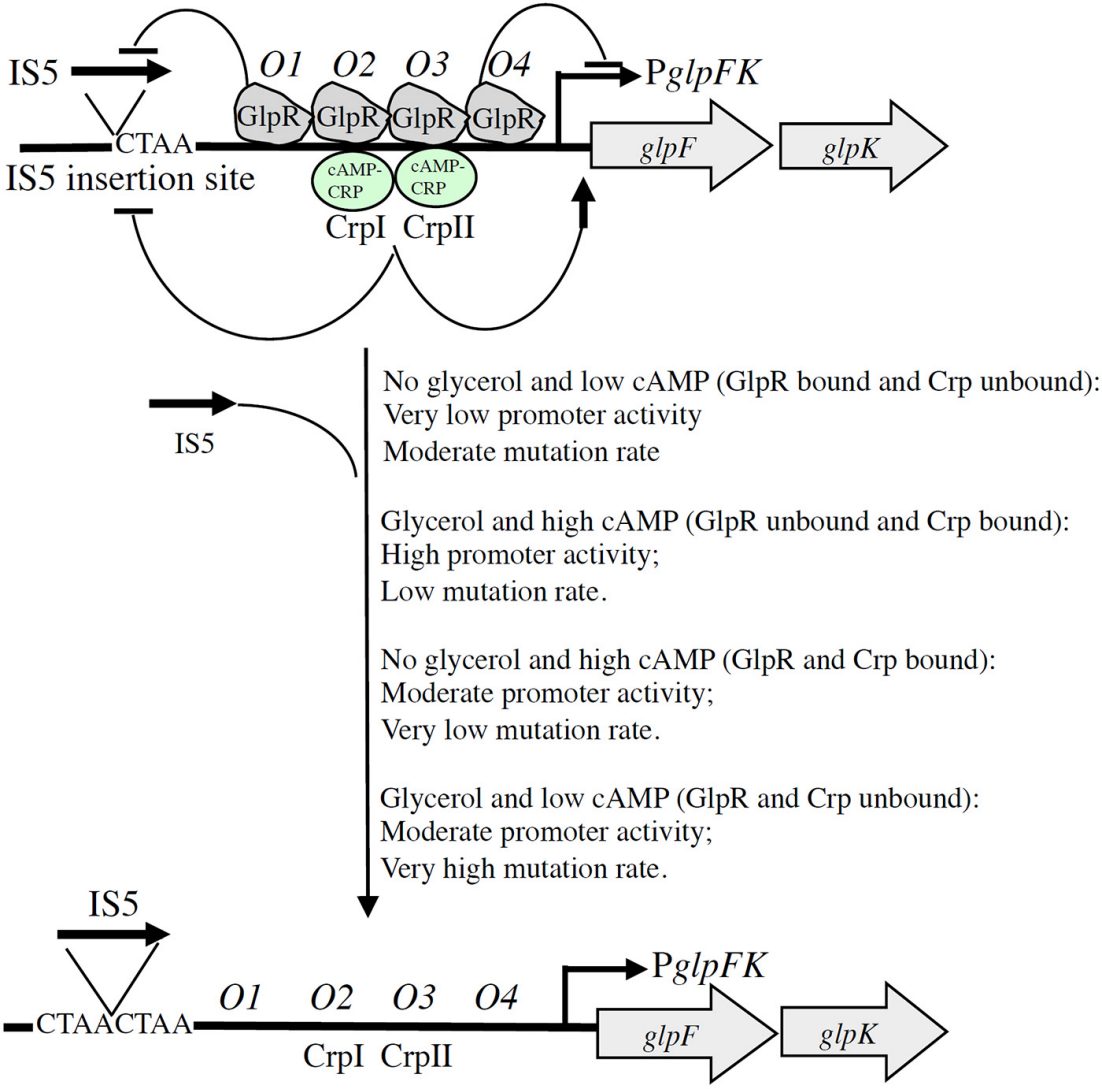
**Schematic diagram illustrating dual GlpR-mediated/cAMP-CRP-mediated control of (right) the level of *glpFK* transcription and (left) the rate of IS5 hopping into the activating site upstream of the *glpFK* promoter (directed mutation)**. With GlpR bound to its operators (*O1–O4*) (in the presence of GlpR and the absence of glycerol), transcription and IS5 hopping both occur at low rates. When GlpR is not bound to its operators (in the absence of GlpR or in the presence of glycerol), both transcriptional initiation and IS5 hopping increase about 10×. Binding of GlpR to operator *O1* preferentially blocks IS5 insertion, while binding of GlpR to operator *O4* preferentially blocks transcription as indicated. Binding of cAMP-CRP to its transcriptional activating sites, CrpI and CrpII, similarly inhibits IS5 hopping even though binding of this complex promotes *glpFK* transcription. Glucose inhibits IS5 insertion by a mechanism independent of glycerol, GlpR, cAMP, and CRP. (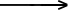, activation; 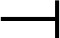, inhibition or repression).

The mechanism of IS5-mediated *glpFK* promoter activation in wild type cells provides relief from starvation when glycerol is present and a cAMP-depressing toxic substance, such as 2-deoxyglucose, is simultaneously present. Such non-metabolizable sugar derivatives are synthesized by many organisms and therefore are present in nature. This example of directed mutation could therefore have been selected for during evolution. It appears to be a genuine example of directed mutation, with mutations arising at a greater rate under conditions that allow benefit to the organism. The fact that mutation rate is influenced by the presence of glycerol in a process mediated by the glycerol repressor, and by cAMP in a process mediated by CRP, provides mechanistic explanations for IS5-mediated directed mutational control. This mechanism, illustrated in Figure 2, allows rationalization of the presence of four GlpR binding sites and two CRP binding sites in the control region of the *glpFK* operon. Our studies also provide the rationale for the evolution of this elaborate mechanism of gene activation.

### Conflict of interest statement

The authors declare that the research was conducted in the absence of any commercial or financial relationships that could be construed as a potential conflict of interest.
